# Multiple-Pathway Analysis of Double-Strand Break Repair Mutations in *Drosophila*

**DOI:** 10.1371/journal.pgen.0030050

**Published:** 2007-04-13

**Authors:** Dena M Johnson-Schlitz, Carlos Flores, William R Engels

**Affiliations:** Department of Genetics, University of Wisconsin, Madison, Wisconsin, United States of America; Stowers Institute for Medical Research, United States of America

## Abstract

The analysis of double-strand break (DSB) repair is complicated by the existence of several pathways utilizing a large number of genes. Moreover, many of these genes have been shown to have multiple roles in DSB repair. To address this complexity we used a repair reporter construct designed to measure multiple repair outcomes simultaneously. This approach provides estimates of the relative usage of several DSB repair pathways in the premeiotic male germline of *Drosophila*. We applied this system to mutations at each of 11 repair loci plus various double mutants and altered dosage genotypes. Most of the mutants were found to suppress one of the pathways with a compensating increase in one or more of the others. Perhaps surprisingly, none of the single mutants suppressed more than one pathway, but they varied widely in how the suppression was compensated. We found several cases in which two or more loci were similar in which pathway was suppressed while differing in how this suppression was compensated. Taken as a whole, the data suggest that the choice of which repair pathway is used for a given DSB occurs by a two-stage “decision circuit” in which the DSB is first placed into one of two pools from which a specific pathway is then selected.

## Author Summary

DNA is a fragile thread that often breaks. When it does, the cell must find a way to splice the broken ends back together in order to continue its cycle of replication. Cells possess an array of ways to rejoin broken DNA ends, each with advantages and disadvantages. Some are “quick and dirty,” sacrificing accuracy for robustness. They do the basic job of resealing the break but often result in random base changes at the site of the repair. At the other extreme are methods with greater fidelity but added restrictions, such as requiring chromosome replication. We used an experimental system to obtain highly accurate measurements of the relative usage of various repair methods in developing germ cells of fruit flies. The measurements were made in normal flies as well as those carrying mutations at each of 11 genes involved in DNA repair. Most previous studies of these genes focused on specific biochemical pathways. Our results looked at how the repair apparatus as a whole compensates for defects in individual components. The data point to a “decision circuit” for matching each break to a repair method and provide new insight into how our DNA repair machinery protects the genome's integrity.

## Introduction

In the last ten years the study of double-strand break (DSB) repair has become more urgent and more difficult. The urgency arises from findings that defects in DSB repair are linked to elevated cancer risks [1] and phenotypes that resemble accelerated aging [2–4]. Meanwhile, the complexity of analyzing DSB repair has increased exponentially with the number of pathways, genes, and interactions that have been discovered [5–7]. Breaks can be repaired via nonhomologous end-joining (NHEJ) [8]or homologous recombination (HR) [9], each of which can be further subdivided into several pathways, all requiring genes for checkpoints, signaling, and effecting the repair itself.

In view of this complexity, it is fortunate that the high degree of conservation in DSB repair systems makes it possible to use model organisms such as *Drosophila* to obtain generalizable results concerning the basic processes. In *Drosophila,* a large-scale screen for mutagen sensitivity has been useful in identifying DSB repair genes [10]. A variety of methods is available for studying DSB repair in *Drosophila*. These include the use of excision of P transposable elements to create breaks [11–23] as well as transplanted endonucleases and recombinases derived from microorganisms [24–27].

The repair reporter construct (Rr3) is designed to yield simultaneous measurements of multiple DSB repair outcomes in the *Drosophila* germline [28]. Other such reporters have been valuable in mammalian systems [29,30] and yeast [31–33]. Measurements obtained with Rr3 reflect the relative usage of NHEJ, single-strand annealing (SSA), and homologous repair with conversion from the homolog (HR-h). They also provide further quantitative information about specific events within these pathways, including the length of conversion tracts, deletion formation, and crossing over [34–36]. Rr3 has been used to show that the relative usage of DSB repair pathways changes with developmental stage [28]. Another surprising finding was that as adult flies age, their usage of HR for repair increases in the germline at the expense of other repair pathways [34,36,37]. Studies with Rr3 also provided evidence that the *Drosophila* version of *BLM*, the Bloom syndrome gene, is needed to resolve double Holliday junctions via dissolution [35].

A major advantage of using Rr3 as opposed to measuring one pathway at a time is that the Rr3 analysis reveals not only which pathway(s) are inhibited by a given condition or genotype, but also which other pathways are used to compensate. This compensation is easily seen as a negative correlation between the relative usage of the various pathways [28] such that the total of the various repair outcomes remains close to 100%. Here we use Rr3 to compare the effects of mutations in 11 genes related to DSB repair. The results provide information concerning how these mutations depress specific pathways, some of which is expected from what is already known about the genes. More interestingly, the results also show how other pathways compensate for each defect. We find that when a given pathway is suppressed, the defect can be compensated in more than one way depending on which mutant gene is responsible for the suppression. This analysis provides a fresh insight into the process by which each DSB is channeled into one of many possible repair outcomes and how the DSB repair system as a whole adjusts when one or more of its components is missing.

## Results

### Measurements of Multiple DSB Repair Outcomes

**Figure 1:**
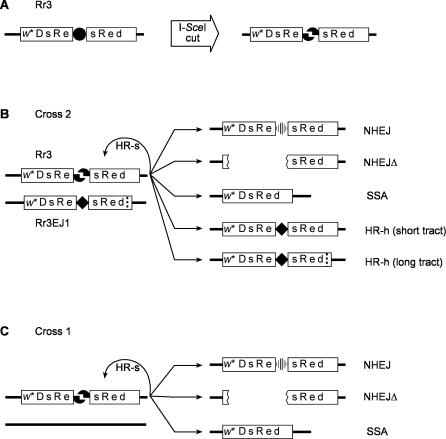
Use of Rr3 to Measure Multiple DSB Repair Pathways Details of the method were published previously [28,34,35]. (A) The Rr3 construct, which is inserted within the *Drosophila* genome, is cut by the rare-cutting endonuclease, I-SceI to yield a DSB. The cut site (•) lies between two parts of a 147-bp direct duplication (sRe) within the red fluorescent reporter gene, DsRed. The transgene is carried within a P element that also has a mini-white (w*) visible marker. The insertion used in these studies is at cytological position 48C. (B) Test males for cross 2 carry Rr3 on one homolog opposite its derivative, Rr3EJ1, on the other. The cut site on Rr3EJ1 is replaced by a 12-bp insertion (solid diamond), and there is a 16-bp deletion located 156 bp to the right and indicated by a dotted vertical line. Repair of this break by one of the available pathways produces recognizably different products that are seen in the next generation. (C) Cross 1 is an alternative version of the Rr3 procedure where the homolog lacks Rr3EJ1. In this case there is no opportunity for repair via HR-h.

The use of Rr3 to measure multiple outcomes of DSB repair has been described in detail elsewhere [28,34–36]. Briefly, DSBs are created at the recognition site of the rare-cutting endonuclease, I-SceI, located within a red fluorescent reporter gene, *DsRed,* and flanked by a direct repeat of 147 bp (Figure 1A). The endonuclease gene, located on another chromosome, is expressed continuously and in all tissues, but we analyze only breaks that occur in the germ cells. Figure 1B shows the five distinguishable outcomes that are observed among the progeny. If repair occurs via conversion with the sister chromatid as template (HR-s), the recognition site is restored, and Rr3 is available for another round of breakage and repair. The cycle can continue until one of the five measured outcomes occurs, all of which destroy the recognition site. We identify these outcomes among the offspring by scoring (i) visible markers and sex to determine the presence of the Rr3-derived chromosome, the endonuclease transgene, and to detect crossing over between flanking markers; (ii) DsRed fluorescence to indicate collapse of the duplication in all or part of the fly; and (iii) single-fly PCR tests in a subset of the offspring to distinguish between specific outcomes. The measured outcomes are:

#### NHEJ.

End-joining usually results in small changes at the breakpoint that inactivate the I-SceI cut site. NHEJ events are scored only among the progeny that also receive the endonuclease gene. This restriction allows us to distinguish them from unchanged Rr3 copies that express DsRed as mosaics following somatic SSA repair. PCR is used on all or a sample of the non-DsRed flies in this group to distinguish NHEJ events from HR-h. The NHEJ frequencies we report do not include the long deletions classified as NHEJΔ below.

#### NHEJΔ.

Infrequently, longer changes occur that inactivate the mini-*white* gene within Rr3 (w* in Figure 1). These events are usually deletions. They are scored phenotypically by eye color among all offspring that receive Rr3.

#### SSA.

Collapse of the 147-bp direct duplication results in constitutive expression of DsRed. These events are scored only among offspring that do not inherit the endonuclease gene to distinguish SSA products from intact Rr3. The latter develop as DsRed mosaics in the presence of endonuclease [28].

#### Short HR-h.

Conversion from the homolog places a recognizable sequence at the breakpoint. Single-fly PCR tests distinguish this outcome from NHEJ. This category includes only those HR-h events whose conversion tract extends less than 156 bp rightward (Figure 1B).

#### Long HR-h.

Same as above, but with a longer conversion tract in the rightward direction, as indicated by the presence of a distinguishing 16-bp deletion that was copied from the template on the homolog and detected in PCR tests (Figure 1B).

Figure 1C shows an alternative version of the Rr3 test in which no template for HR-h is present. This procedure is called cross 1 as opposed to cross 2 shown in Figure 1B. We discuss cross 2 first to emphasize its role as our primary source of information. The value of cross 1 is in determining the relative usage of NHEJ and SSA when HR-h is unavailable, thus providing further information on compensation among pathways. In addition, cross 1 does not require PCR tests, thus permitting larger sample sizes.

We used crosses 1 and 2 to measure DSB repair outcomes in 30 genotypic backgrounds including mutations at 11 DSB repair loci. The results are in Table 1. Information about the repair genes and our interpretations of the results are in Table 2. The 11 loci were selected to include a range of repair functions. Some of these genes have been studied extensively and others only minimally. All of these genes have orthologs, and many belong to families that have more than one name. Here we have attempted to use the most widely recognizable name for each gene, but we have provided the other names for reference in Table 2.

**Table 1:**
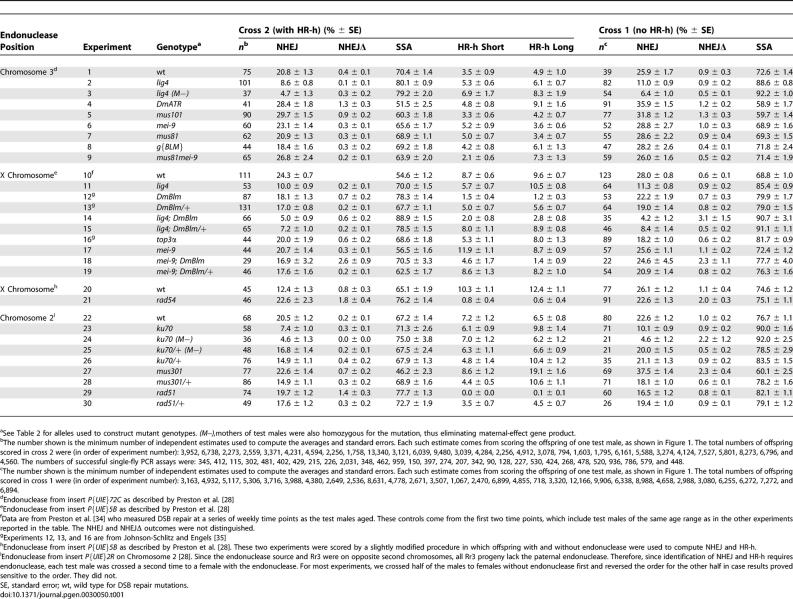
Measurements of DSB Repair Outcomes from Rr3

**Table 2:**
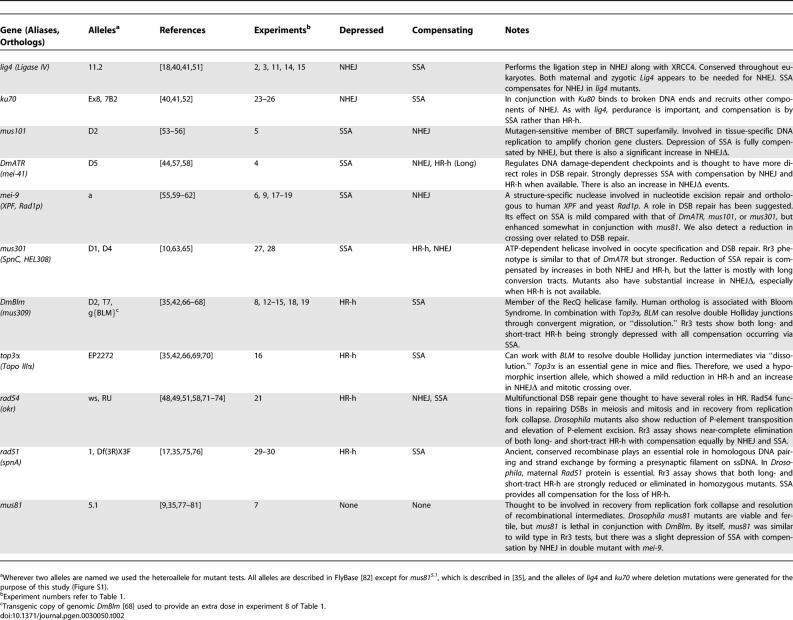
DSB Repair Genes

Standard errors are given in Table 1 for all measurements to provide an indication of their precision. However, these standard errors were not used for hypothesis testing that was performed with distribution-free procedures as described in Materials and Methods. The experiments are shown in four groups such that those within each group have the same endonuclease source and the same scoring techniques. Inferences about the mutations are drawn by comparing their measurements to control values, either wild type or heterozygotes, within the same endonuclease group.

### Compensation between Pathways

**Figure 2:**
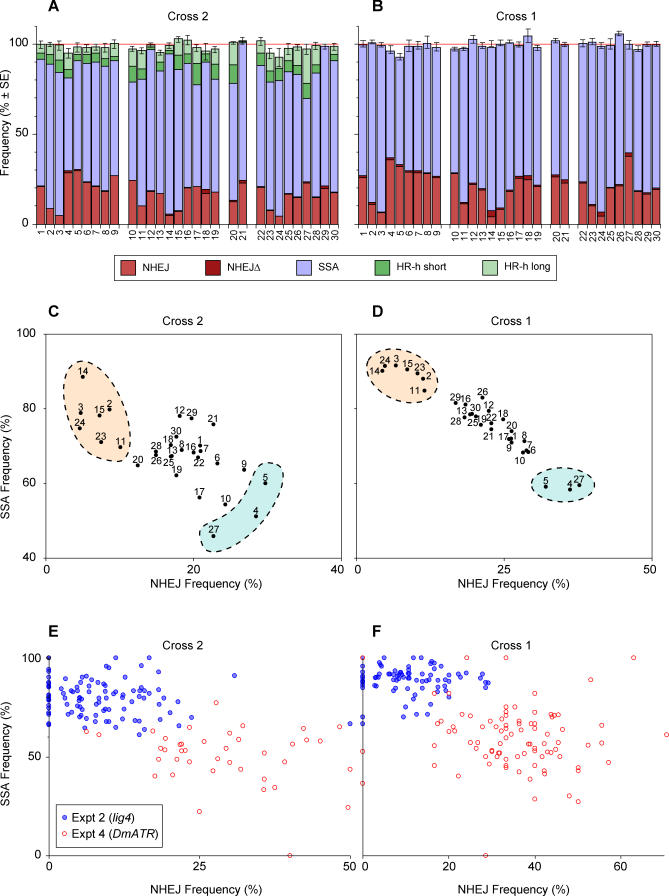
Compensation between Pathways (A) The results of the cross 2 tests in Table 1 are plotted as stacked columns to show that the sum of the five outcomes remains at approximately 100% despite wide variation in the individual proportions. To obtain standard errors for the sums, we identified all single-male crosses for which the frequencies of all outcomes could be estimated. We then summed the estimates for each male and used the empirical standard error of those independent totals. (B) is the same as (A) except for cross 1 data where only NHEJ, NHEJΔ, and SSA can be measured. (C and D) Show scatterplots of SSA versus NHEJ for each of the experiments in Table 1. The strong negative correlations show compensation between SSA and NHEJ. Cross 2 data are in (C) and cross 1 data are in (D). The two outcomes, SSA and NHEJ, were selected for these plots because they were measured independently among the offspring of each male. NHEJ estimates are derived only from the offspring that received the endonuclease, and SSA came from those that did not. Highlighted regions indicate experiments showing strong compensation by SSA for a defect in NHEJ or the reverse. In (E and F) each dot represents estimates of NHEJ and SSA for a single test male. Data for experiments 2 and 4 are shown, which test *lig4* and *DmATR* mutations. Cross 2 data are shown in (E) and cross 1 in (F). Correlations for each of the four sets of points were negative and statistically significant for the cross 2 sets (*p* = 0.047 and *p* = 0.006 for experiments 2 and 4, respectively, one-tail).

One indication that there is compensation between DSB repair pathways is that the sum of the outcomes remains close to 100% across experiments despite wide variation in individual frequencies. This property can be seen in Figure 2A and 2B where the five measurements from cross 2 and the three from cross 1 are plotted as stacked columns. We see that the total column heights are relatively constant, staying within the range 93%–103% for cross 2 and 93%–106% for cross 1 even though some of the individual measurements vary over a much wider range. It should be noted that there is no artificial constraint on this sum, since the SSA frequency was computed using a different subset of the offspring from those used to compute NHEJ and HR-h.

Another way to see compensation between pathways is by the negative correlation between individual outcomes. The frequency of SSA for each of the 30 experiments is plotted against NHEJ for cross 2 in Figure 2C and for cross 1 in Figure 2D. Those two outcomes were selected because, as mentioned above, they are computed from different subsets of the offspring, and because they are available from both crosses. A strong negative correlation is apparent from both plots. Note also the consistency between the two crosses at the extreme ends of the distribution (highlighted regions). Experiments 2, 3, 11, 14, 15, 23, and 24 appear at the upper left ends of both plots. These are the experiments with mutants at the *lig4* or *ku70* locus. Our interpretation, as discussed below, is that these two loci depress NHEJ and are compensated by SSA. At the lower right ends of both scatterplots lie experiments 4, 5, and 27, which represent mutations at *DmATR, mus101,* and *mus301*. These three mutations are apparently defective in SSA with compensation by NHEJ. However, as discussed below, these three loci differ from each other by whether they can also be compensated by HR-h.

Finally, one can detect compensation through the negative correlation between measurements in individual males. As reported previously (see Figure 6A in [28]), random differences in DSB repair pathway usage between individuals can also display compensation. Figure 2E and 2F show this effect for crosses 1 and 2 with the test males from experiments 2 and 4.

### Mutations That Suppress NHEJ: *lig4* and *ku70*

**Figure 3:**
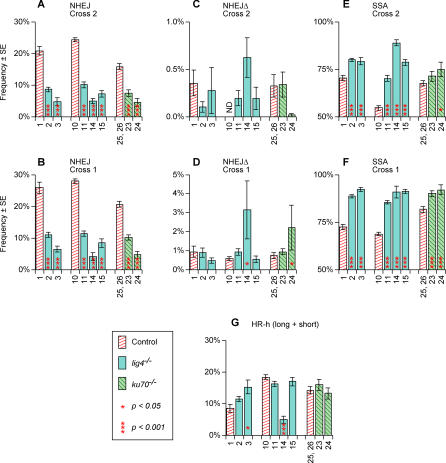
DSB Repair Frequencies for *lig4* and *ku70* and Their Controls Experiment numbers, frequency estimates, and standard errors are from Table 1 except that HR-h frequencies were computed by first summing the long- with short-tract HR-h frequencies for each individual test male, then computing averages and standard errors. Significance values were computed as described in Materials and Methods. All comparisons are between a mutant genotype and its corresponding control. That is, experiments 2 and 3 are compared with experiment 1; experiments 11, 14, and 15 with 10, etc. Experiments 25 and 26, the heterozygous controls for *ku70*, were pooled since they were not significantly different from each other. NHEJΔ was not measured in experiment 10, cross 2 and the NHEJ value for that control includes NHEJΔ events. However, since the NHEJΔ frequency in other controls (C) were less than 0.5%, this difference is not expected to affect the use of experiment 10 as the NHEJ control for experiments 11, 14, and 15 in (A). Shown are: (A) NHEJ cross 2; (B) NHEJ cross 1; (C) NHEJΔ cross 2; (D) NHEJΔ cross 1; (E) SSA cross 2; (F) SSA cross 1; (G) HR-h cross 2 with long- and short-tract events pooled.

The data in Table 1 and Figure 2 show that certain genotypes display conspicuous suppression of NHEJ. Experiments 2, 3, 11, 14, and 15, which test null mutants for *lig4,* show reduction in NHEJ usage from 2- to 6-fold relative to the corresponding controls. Figure 3A shows the total cross 2 NHEJ frequencies and their controls, and Figure 3B shows the same results for cross 1. All ten of these comparisons—including five in cross 2 and five in cross 1—showed a highly significant reduction in NHEJ frequency. We conclude that NHEJ repair is reduced to less than half of its normal usage in *lig4* mutants.

Similarly, *ku70* mutants were tested in experiments 23 and 24. Figure 3A and 3B compare their NHEJ frequencies to their heterozygous controls from experiments 25 and 26. These mutants also showed a clear reduction in NHEJ usage, with all four comparisons being highly significant. Therefore, *ku70* mutants are similar to *lig4* in lacking most of the normal NHEJ repair capability.

The results also show a maternal effect contribution affecting NHEJ for both *lig4* and *ku70.* By comparing experiment 2 with 3, we see significantly less NHEJ usage when *lig4* mutant males had mutant mothers in cross 2 (*p* = 0.009) (Figure 3A) and in cross 1 (*p* = 0.004) (Figure 3B). The *ku70* mutant males also showed less NHEJ usage when their mothers were *ku70^−/−^* by comparing experiments 23 and 24 in crosses 2 and 1 (*p* = 0.070 and *p* = 0.018, respectively) (Figure 3A and 3B). The *ku70* mutant test males from cross 1 of experiment 24 also showed mosaicism in their eye color, which was not present in any other class. We interpret this mosaicism as a result of somatic NHEJΔ events. In addition to the results in Table 1, we also used cross 1 to analyze test males with an extra copy of *Ku70,* but no differences relative to the controls were detected.

Despite the pronounced drop in “normal” end-joining repair for *lig4* and *ku70* mutants, there was no such decrease for the end joining accompanied by long deletions (Figure 3C and 3D). In fact, experiments 14 and 24 showed significant increases in NHEJΔ (*p* = 0.002 and *p* = 0.043, respectively) for cross 1. These deletion events could result when the more typical NHEJ process fails.

Unexpectedly, we found that the reduction in NHEJ frequencies in *lig4* and *ku70* mutants was entirely compensated by an increase in SSA, as seen in Figure 3E and 3F. All ten of the relevant comparisons for *lig4* were highly significant, and three of the four comparisons for *ku70* were also significant. In contrast, there was little or no compensation by HR-h, as shown in Figure 3G. In fact, four of the seven comparisons actually show a decrease in HR-h frequency relative to their controls, although in the case of experiment 14 this decrease is most likely a result of the *DmBlm* mutation also present in that genotype. We conclude that SSA provides all or nearly all of the compensation for the loss of NHEJ in *lig4* and *ku70* mutants. It should be emphasized that this conclusion applies only to breaks within the Rr3 construct where an opportunity for SSA is provided via the 147-bp duplication. The finding that SSA but not HR-h can compensate for the reduction in NHEJ in this class of mutations suggests that there is a pool of DSBs that can “choose” between NHEJ and SSA, but not HR-h. We shall refer to this hypothetical pool of breaks as pool 1 in subsequent discussions.

### Mutations That Suppress HR-h: *DmBlm, top3α, rad54,* and *rad51*

**Figure 4:**
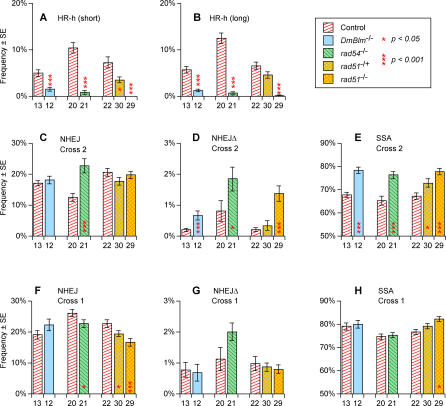
DSB Repair Frequencies for *DmBlm, rad54,* and *rad51* Experiment numbers, frequency estimates, and standard errors are from Table 1. Significance levels (all two-tailed) were computed by permutation tests as described in Materials and Methods. Each comparison was performed with data from a mutant genotype and its corresponding control as in Figure 3. Shown are: (A, B) HR-h cross 2; (C) NHEJ cross 2; (D) NHEJΔ cross 2; (E) SSA cross 2; (F) NHEJ cross 1; (G) NHEJΔ cross 1; (H) SSA cross 1.

HR-h was significantly reduced in the homozygotes of *DmBlm, rad54,* and *rad51* as well as in the heterozygotes of *rad51*. These effects are seen in Table 1 and Figure 4A and 4B. The most pronounced reduction was in the *rad51* homozygotes that reduced long-tract HR-h more than 100-fold, and eliminated short HR-h completely. Null homozygotes of *DmBlm* and *rad54* also reduced both long- and short-tract HR-h (Figure 4A and 4B and [35]). Homozygotes for a weak hypomorphic allele of *top3α* behaved in a qualitatively similar way to *DmBlm*, as reported previously [35].

Interestingly, *rad54* did not behave analogously to the other HR-suppressing mutants in terms of how the reduction in HR-h was compensated by other pathways. The *rad54* homozygotes showed significant increases in both SSA (Figure 4E) and NHEJ (Figure 4C) whereas SSA provided essentially all of the compensation in the *DmBlm* and *rad51* mutants (Figure 4C and 4E). The existence of two mutations, *DmBlm* and *rad51*, where suppression of HR-h is compensated solely by SSA, suggests that there is a pool of DSBs where the choice of repair pathways is limited to HR-h and SSA. We shall refer to this hypothetical group of breaks as pool 2 in discussions below.

This compensatory increase in SSA was pronounced in cross 2 but weak or nonexistent in cross 1 where there was no opportunity for HR-h (Figure 4E versus 4H). This relative lack of effect in cross 1 implies that the primary phenotype of these mutants is the drop in HR-h, whereas the concomitant increases in SSA seen with all three mutants may be considered an indirect effect. The smaller cross-1 increase in SSA seen in homozygous *rad51* mutants (Figure 4H) can be interpreted as an indirect consequence of a reduction of conversion off the sister chromatid, HR-s. This interpretation relies on the “decision circuit” model discussed below.

All four mutant types showed an increase in long-deletion NHEJΔ repair (Figure 4D and [35]). However, the overall frequency of these deletion events was insufficient to provide substantial compensation for the lack of HR-h. Instead we suggest that the increase in long deletions is the result of aberrant HR-h repair. Consistent with this hypothesis, note that the increase in deletions occurs even in the *DmBlm* and *rad51* mutants where normal NHEJ is not enhanced. In addition, there were no significant changes in NHEJΔ frequencies in cross 1 where HR-h cannot occur (Figure 4G).

In addition to the measures reported in Table 1, we also recorded the frequency of crossing over in cross 2 between markers flanking Rr3 for all 30 genotypes shown in the table. These values are not included in the table, because the only significant differences relative to the controls occurred in the *DmBlm* and *top3α* mutants that were reported previously [35].

Finally, Figure 4F shows that *rad54* and *rad51* mutants both showed a decrease in NHEJ in cross 1. This effect stands in contrast to the result in cross 2 where *rad51* had no effect on NHEJ and *rad54* actually had a highly significant increase (Figure 4C). In the case of *rad51* this difference between crosses 1 and 2 can be explained in terms of the “decision circuit” model discussed below.

### Mutations That Suppress SSA: *mei-9, DmATR, mus101,* and *mus301*

**Figure 5:**
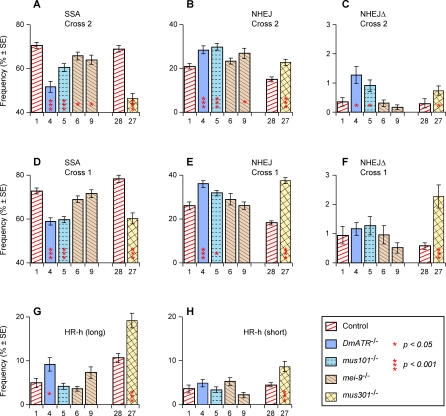
DSB Repair Frequencies for *DmATR, mus101, mei-9,* and *mus301* Experiment numbers, frequency estimates, and standard errors are from Table 1. Significance levels (all two-tailed) were computed by permutation tests as described in Materials and Methods. Each comparison was performed with data from a mutant genotype and its corresponding control as in Figure 3. Shown are: (A) SSA cross 2; (B) NHEJ cross 2 (C) NHEJΔ cross 2; (D) SSA cross 1; (E) NHEJ cross 1; (F) NHEJΔ cross 1; (G) HR-h (long) cross 2; (H) HR-h (short) cross 2.

We found that SSA was suppressed in mutants for *DmATR, mus101, mus301,* and, to a lesser extent, *mei-9*. This suppression is apparent in both crosses, as seen in Figure 5A and 5D. The suppression of SSA was highly significant in a statistical sense, but note that none of the mutant genotypes reduced SSA to less than 67% of its control value. This result contrasts sharply with the mutants that suppress NHEJ and HR-h, sometimes reducing these outcomes to small fractions of their normal values (Figures 3 and 4).

The effect of *mei-9 (Rad1p)* in suppressing SSA is only apparent in experiments 6 and 9 where the endonuclease is supplied from insertion ubiquitin-driven I-SceI endonuclease (UIE)-72C on chromosome 3 and not in experiments 17, 18, or 19 where the endonuclease source is insertion UIE-5B on the X chromosome. As noted previously [28], different endonuclease sources can result in subtle differences in the timing and frequency of DSB formation. Such differences are more likely to be critical in the case of *mei-9* whose effect on SSA is relatively weak.

All four mutations utilized NHEJ to compensate for the drop in SSA. The increase in NHEJ was significant for *DmATR, mus101,* and *mus301* in both cross 2 (Figure 5B) and cross 1 (Figure 5E) and for *mei-9* in cross 2 (experiment 9, Figure 5B). In addition, *DmATR* and *mus301* also used HR-h to compensate (Figure 5G and 5H), whereas no such compensation occurred in mutations for *mus101* or *mei-9*. This compensation by HR-h occurred primarily by long-tract conversion events (Figure 5G) rather than short-tract HR-h that was only significant for *mus301* (Figure 5H). One possible explanation for this specificity is that some DSBs, which would have been repaired via SSA were it not for the lack of *DmATR* or *mus301* function, had undergone extensive gap widening before being repaired via HR-h.

We also observed a significant increase in the NHEJΔ outcome for *DmATR* and *mus101* in cross 2 (Figure 5C) and for *mus301* in both crosses (Figure 5C and 5F). Overall, the list of mutations that showed an increase in deletion formation includes both mutations that suppress NHEJ *(lig4* and *ku70)*, all four of those that suppress HR-h *(DmBlm*, *top3α, rad54,* and *rad51)*, and three of the four that suppress SSA *(DmATR, mus101,* and *mus301)*. No mutation showed a decrease in NHEJΔ frequency. We conclude that NHEJΔ can be increased by a wide range of mutational changes but not reduced. We interpret this finding to indicate that failure of repair via any of the pathways can result in long deletions, but that such deletions are not formed in the normal course of any pathway.

### Other Mutant Phenotypes

We performed further tests on some of the mutants in addition to the Rr3 assays in Table 1. For five of the loci we measured crossover frequencies related to DSB repair at an I-SceI cut site other than Rr3. This cut site, located at cytological position 50C and described previously [35], differs from Rr3 by the absence of a flanking duplication suitable as a substrate for SSA repair. Therefore, HR-h competes only with NHEJ. The results (Figure S2) showed crossing over in *mei-9* mutants was reduced to less than one-fourth the control levels. The other mutants tested, *lig4, mus81, DmATR,* and *mus101,* showed no significant difference from the controls. Our interpretation is that *mei-9* mutations are defective for some aspect of the repair process in which double Holliday junctions are formed and resolved via strand exchange as opposed to dissolution. The relative rarity of crossing over during DSB repair in *Drosophila* suggests that this sequence of events is not a major pathway in wild-type flies.

We tested the *ku70^EX8^* deletion shown in Figure S1 to verify that *ku70* was mutated and to characterize other effects of the mutation. Figure S3 shows that homozygotes for this deletion are hypersensitive to the chemical mutagen methyl methanesulfonate and that this sensitivity is rescued by a 13-kb *Drosophila Ku70* transgene [38]. Similarly, Figure S4 shows the same homozygotes may also be mildly hypersensitive to gamma irradiation and that the transgene also restores them to normal sensitivity. Finally, we noticed that many of the *ku70^EX8^* homozygotes had an excess of macrochaetae (large thoracic bristles) that were deformed or reduced in size, and this phenotype was rescued by *Ku70* transgenes (Figure S5). This macrochaetae phenotype may be similar to that seen by Brodsky et al. in mutants of *mus304* [39]. The authors of that study interpreted the phenotype as the result of somatic mosaicism for haploinsufficiency at any of the *Minute* loci owing to excess production of deletions during development. This interpretation is consistent with the excess production of NHEJΔ deletions seen in our *ku70* mutant tests (Figure 3D).

## Discussion

### The Value of Measuring Several Pathways at Once

Comparison of the relative usage of DSB repair pathways has been valuable in understanding oncogenic events in mammals [29,30] and screening for repair mutations in yeast [32]. We have made use of the Rr3 assay to detect changes in the mix of DSB repair pathway usage during development [28], to identify age-related changes [34] and to analyze the effects of specific repair loci [35]. Here we apply the approach to a broad spectrum of repair mutations to compare their effects on the DSB repair system as a whole.

By measuring multiple repair outcomes from a single pool of DSBs we can gather information that would be more difficult to obtain with an experimental system in which only one type of outcome is measured. For example, McVey et al. [18] found that *Drosophila lig4* mutants had no significant drop in NHEJ repair, whereas our data show up to a 4-fold decrease (Figure 3A and 3B). Several factors could contribute to this discrepancy. The enhanced sensitivity obtained when each measured DSB repair outcome is compared directly with competing outcomes provides a clear benefit for the Rr3 approach. Other differences are the nature of the endonucleases, P transposase versus I-SceI, and that the breaks in Rr3 can be repaired by SSA, which may allow recovery of some DSBs that would otherwise have been lost through cell death.

The Rr3 analysis provides two pieces of information about each mutation: which pathway(s) are suppressed and which are used for compensation (Table 2). By classical genetic inference, suppression of a pathway in a mutant is taken to indicate a primary role of the gene. For some of the genes in our study, this suppression was predictable from previous work. For example, our finding that *lig4* and *ku70* both reduce NHEJ (Figure 3A and 3B) is not surprising given that both genes have long been linked to NHEJ repair [40,41]. Similarly, the loss of HR-h in mutations at the *DmBlm, Rad51, Top3α,* and *Rad54* loci (Figure 4A and 4B; Table 1) was expected from previous work on these genes [6,42], as was the decrease in SSA in *DmATR* and *mei-9 (Rad1p)* mutations [43,44]. On the other hand, suppression of SSA by mutations at *mus101* and *mus301* (Figure 5A and 5B) was not apparent from any previous knowledge about the functions of these genes, and the roles of these two genes in SSA remains to be determined.

Of the 11 DSB repair loci in this study, ten of them suppressed exactly one of the three pathways (NHEJ, SSA, and HR-h), and none reduced more than one. The only mutation with no differences from wild type in the Rr3 assay was *mus81*, although synthetic lethality in the *mus81-DmBlm* double mutant [35] suggests a role for *mus81* in DSB repair and/or recovery from replication fork collapse in *Drosophila*. Our finding that no mutation suppressed more than one pathway is perhaps surprising in view of the multiple roles found for many DSB repair genes. This apparent simplicity need not be taken to imply that each gene has a major role in only one pathway, but rather may be interpreted to reflect the robustness of the overall DSB repair system and its ability to compensate for defects in any one component.

The second piece of information from the Rr3 analysis reveals how each defect is compensated by increases in usage of other pathways. This information opens a new dimension in which to compare DSB repair phenotypes. For example, the finding that *lig4* and *ku70* mutations are compensated by SSA and not HR-h (Figure 3E–3G) could not have been predicted from previous data. Furthermore, there are cases in which mutations in two genes knock down the same pathway, but differ from each other in how the effect is compensated. For example, *rad54, DmBlm,* and *rad51* mutants all cause severe reduction in HR-h repair, but *rad54* is compensated by increases in both SSA and NHEJ whereas *DmBlm* and *rad51* are compensated only by an increase in SSA (Figure 4). Another example is that of *mus101* and *mei-9* compared with *DmATR* and *mus301.* All four mutations reduce the SSA outcome, but the first two are compensated only by an increase in NHEJ whereas the latter two are associated with increases in both NHEJ and HR-h (Figure 5). Furthermore, the HR-h component of this compensation occurs primarily with conversion tracts longer than 156 bp rightward (i.e., HR-h long). Differences such as these provide new insights into the complex process by which each DSB is channeled to one of the available repair pathways. In particular, as elaborated below, we suggest that how a given defect is compensated depends on where the defect lies within a stepwise decision process, as opposed to a biochemical pathway, in which each DSB is ultimately handled by one repair pathway.

### The Nature of Compensation

Compensatory changes in DSB repair pathway usage are clear from the negative correlations among Rr3 outcomes seen in Figure 2 and elsewhere in this report, as well as from previous publications [28,34–36], but the underlying basis of this compensation is less clear. One possibility is that unsuccessful repair attempts are selectively eliminated, thus increasing the relative frequency of the successful repair processes among the survivors. *Drosophila* males can produce a sufficient excess of sperm to accommodate considerable selection without detectable loss of fertility. This selection could occur premeiotically, as through apoptosis. It could also occur through formation of aberrant repair products that survive gametogenesis but are eliminated as “dominant lethals” postmeiotically. This latter possibility is most easily tested, as it would result in decreased recovery of the Rr3 chromosome relative to its homolog. We found no evidence for such an effect. For example, in experiment 1 a total of 3,952 progeny carried Rr3 versus 4,630 with Rr3EJ1, giving a recovery frequency for Rr3 of 46.0% in the absence of any DSB repair mutation. This frequency may be compared with experiment 2 where the *lig4* mutation was associated with a marked loss of NHEJ events and compensation by SSA. If the observed decrease in NHEJ from 20.8% in experiment 1 to 8.6% in experiment 2 (Table 1) is to be explained by postmeiotic selection, one would expect the recovery frequency of Rr3 to be reduced to 40.4% in experiment 2. Instead, we found it was nearly identical to the controls: 45.8 ± 0.8% out of a total of 14,724 progeny. A permutation test to compare experiments 1 versus 2 using the Rr3 frequencies among the progeny of each individual male gave *p* = 0.416. We conclude that postmeiotic selection accounts for little or none of the observed compensation.

In the case of premeiotic selection, a testable prediction is that when one type of outcome is knocked down by mutation, all others would increase proportionately. That is, compensation would occur through an increase in all measured outcomes except those directly affected by the mutation. Instead, we found that compensation usually occurred through only one of the alternative outcomes (Table 2). In fact, seven of the ten loci where one outcome was diminished displayed compensation in only one other outcome. Furthermore, one of the remaining three loci *(DmATR)* showed compensation by NHEJ and HR-h, but the increase in HR-h occurred primarily with long-tract conversion events. One could argue that these eight genes have secondary effects on some of the other outcomes, but it would be necessary to postulate that these secondary effects counterbalance the increase just enough to leave a net result of no significant difference from the control.

From the above observations, we conclude that selection, either post- or premeiotic, is unlikely to account for more than a small proportion of the observed compensation. Instead, we suggest that these compensatory changes reflect the process by which the DSB repair mechanism as a whole channels each break into one of the available pathways.

### A “Decision Circuit” for DSB Repair

Our finding (Figure 3) that the loss of most NHEJ repair in *lig4* and *ku70* mutants is compensated only by SSA and not HR-h suggests the existence of a pool of DSBs for which SSA and NHEJ are the only options. A second line of evidence for pool 1 is provided by the mutations *mus101* and *mei-9* in which the reduction in SSA is compensated by an increase in NHEJ but not HR-h (Figure 5).

**Figure 6:**
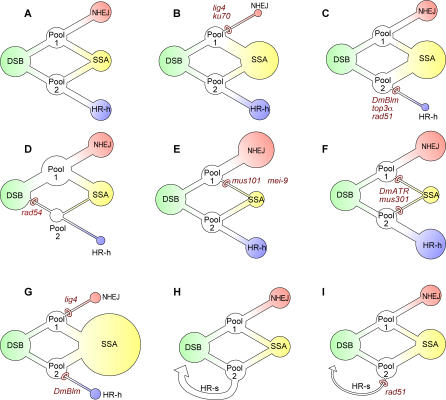
Decision Circuit for Breaks in Rr3 (A) The decision process is represented as distinct from any specific biochemical pathway. It is envisioned as proceeding from left to right. The first step is the placement of each DSB into one of two intermediate pools, labeled pool 1 and pool 2. Breaks in pool 1 can be repaired by either NHEJ or SSA, whereas those in pool 2 are handled by either HR-h or SSA. In addition, HR-s (not drawn) restores the I-SceI cut site, enabling another round of DSB formation and repair. (B–G) Hypothesized effects on the decision circuit by mutant genotypes are shown. When one of the transitions in the circuit is inhibited by reduction of a gene product, use of the alternative route is increased. The sizes of circles surrounding the three measured outcomes, NHEJ, SSA, and HR-h, are meant to reflect the relative frequencies. No attempt was made to scale these circles precisely to the data, since each diagram represents several experiments and sets of estimates. (H) Depicts decision circuit for cross 1, which differs from cross 2 by the absence of any opportunity for HR-h. The HR-s repairs are shown explicitly here and assumed to come from pool 2. (I) Hypothesized effect of *rad51* mutation in cross 1 is shown. Decreased HR-s repair results in more use of SSA. Since only two outcome types are measured in cross 1, the relative frequency of NHEJ is reduced through increased competition with SSA.

The mutations at *DmBlm, top3α,* and *rad51* suggest the existence of a second pool of DSBs. These mutants have reduced HR-h, which is compensated only by SSA and not NHEJ (Figure 4) [35], suggesting that these mutations act upon a subset of breaks to which only NHEJ and SSA are available. We refer to this hypothetical set of DSBs as pool 2. Combining these observations leads us to suggest that breaks in Rr3 are channeled into one of these three repair types via a “decision circuit” as shown in Figure 6A. In this scheme, end-joining outcomes are derived only from breaks in pool 1 while HR-h outcomes come only from pool 2. SSA outcomes, however, can be derived from either pool.

Figures 6B–6F show how ten of the mutant loci in our study are interpreted by this scheme. Each mutation restricts one of the decision options (or two of the options in the cases of *DmATR* and *mus301*), which results in increased usage of the alternative option. For example, the *rad51* mutation is hypothesized to prevent most DSBs in pool 2 from being repaired via HR-h, resulting in such breaks being handled by SSA (Figure 6C). However, since pool 1 is not affected by *rad51,* there is no change in the usage of NHEJ. This description fits the observed phenotype of *rad51* (Figure 4). That is, we saw a severe reduction in HR-h, which was compensated fully by an increase in SSA, while NHEJ remained unchanged.

The decision diagram in Figure 6A was originally drawn solely to visualize the compartmentalization of DSBs into the two hypothetical pools and to provide an interpretation for the single-mutant data as in Figures 6B–6F. However, an additional benefit of this scheme is that it makes two predictions that are met by existing data. First, the representation for single mutants of *lig4* (Figure 6B) and *DmBlm* (Figure 6C) imply that the double mutant would have a greater frequency of SSA than either single mutant, since DSBs in both pools 1 and 2 are expected to utilize SSA more frequently. This expectation is shown in Figure 6G. The *lig4; DmBlm* double mutant was tested in experiment 14 (Table 1) resulting in an SSA frequency of 89% in cross 2, the highest of any of the experiments and in good agreement with the model.

A second prediction is that mutants with reduced SSA will show a milder degree of reduction compared to those that reduce the other two outcomes. This is because SSA outcomes are drawn from both pools of breaks whereas NHEJ and HR-h each draw DSBs from only a single pool. Consistent with this prediction, we found that in the most severe restriction of SSA (by *mus301* in cross 2) the frequency was 67% of its heterozygous control (experiments 27 and 28). In contrast, the other two repair outcomes, NHEJ and HR-h, displayed much more severe reductions in the corresponding mutants: *lig4* mutants without maternal product produced only 22% as much NHEJ as their controls (experiments 3 and 1), and *rad51* homozygotes had less than 1% of the short- or long-tract HR-h frequencies as their controls (experiments 29 and 22). These observations provide a good fit to the model, although other explanations are possible.

Cross 1 can also be represented by a decision circuit diagram, as in Figure 6H. HR-h is not available in cross 1, and conversion from the sister chromatid (HR-s) yields a regenerated I-SceI cut site that is vulnerable to another round of DSB formation. HR-s would also restore the cut site in cross 2, but this event is not shown explicitly in Figure 6A–G. The scheme in Figure 6H provides an explanation for the peculiar behavior of *rad51* mutants that cause a reduction of NHEJ in cross 1 but not cross 2 (Figure 4F versus 4C) while increasing SSA in both crosses (Figure 4E and 4H). We interpret these cross-1 effects as indirect consequences of a reduction in HR-s usage. We cannot measure HR-s directly, as it regenerates the original Rr3 structure. However, as shown in Figure 6I, a reduction in HR-s from pool 2 would enhance usage of SSA. Since NHEJ is the only other outcome from cross 1, its frequency would then decrease owing to elevated competition with SSA. This reasoning, however, applies only to *rad51* and not *rad54,* since the results from cross 2 place the action of *rad54* at a different point in the decision circuit (Figure 6D). The reduction in NHEJ from *rad54* in cross 1 is only weakly significant (*p* = 0.04) and could be spurious. In addition, we do not see any effect on SSA or NHEJ in cross 1 for *DmBlm* or *top3α* even though they are postulated to act at the same segment of the decision circuit as *rad51* (Figure 6C). The reason may be that *DmBlm* and *top3α* have a weaker reduction in HR-h than *rad51,* so their secondary effect on cross 1 (indirectly via HR-s reduction) would be less apparent.

### Physical Basis of the Decision Circuit

At minimum, the diagrams in Figure 6 provide a useful, albeit abstract, way to encapsulate the main points of a complex set of experiments. In discussing these diagrams, we were careful to avoid terms such as “pathway,” which could be taken to imply that the diagrams represent specific biochemical steps. It is also important to emphasize that any process represented by these diagrams is strictly applicable only to breaks formed in the Rr3 construct. Most naturally occurring DSBs lack the direct duplication needed for SSA repair. Furthermore, specific parameters in the design of Rr3, such as the length of its direct duplication (147 bp), are likely to influence the quantitative and even qualitative phenotypes. For example, we found that *rad54* increased usage of SSA for duplications of moderate length, as in Figure 4E, but it actually decreased the SSA outcome or similar repair products for much longer duplications (unpublished data). Finally, the genomic location of Rr3 in these experiments, cytological position 48C, is sufficiently far from the telomere to rule out the breakage-induced replication pathway [45].

We also emphasize that the representations in Figure 6 entail some simplifications of the data. In particular, the five measured outcomes (Figure 1B) are reduced to three, with the short- and long-HR-h outcomes combined and the NHEJΔ outcome not represented. This simplification masks some potentially important details, such as the finding that the reduction in HR-h by mutations at *DmBlm, rad51,* and *rad54* (Figure 6C and 6D) included both short- and long-tract HR-h equally (Figure 4A and 4B), whereas the increase in HR-h effected by *DmATR* and *mus301* (Figure 6F) applies primarily to the long-tract HR-h outcome (Figure 5G and 5H).

Despite the above limitations, it is hard to resist some speculation about the physical basis underlying these diagrams, especially the nature of the hypothesized pools 1 and 2. We can think of pool 1 as containing DSBs for which HR-h (and possibly HR-s) is not available. This restriction could result from the timing of the break within the cell cycle, as recently reviewed [46]. Thus, pool 1 could represent breaks that occurred in G1 or early S phase where HR-s is unavailable, and HR-h is infrequent. Interestingly, the route from “DSB” to “pool 1” in our decision circuit scheme is the only one of the six routes in the diagram that is not reduced by any of the mutations studied, suggesting it may not be under genetic control. That suggestion is consistent with the interpretation that entrance into pool 1 in wild type individuals could be determined by the stage of the cell cycle rather than any enzymatic reaction. An alternative interpretation for pool 1 is that it represents breaks for which pairing with a potential template has not occurred. In that case, SSA and NHEJ remain available, but not HR-h or HR-s.

Pool 2 consists of DSBs that are slated to be repaired by conversion (HR-h and HR-s) or SSA, both of which have extensive 5′ resection of the broken ends as a prerequisite [6,47]. Therefore, resection may be the defining characteristic of pool 2. According to the interpretation in Figure 6D, *rad54* restricts entry into pool 2 suggesting it has an early role in the decision process, acting at or prior to the resection stage. This example serves to emphasize the distinction between the decision process visualized in Figure 6 where *rad54* appears to act early, versus the biochemical repair process itself where there is evidence that *rad54* has one or more later roles [48,49].

### Conclusions

The apparatus for repairing double-strand breaks is ancient and essential. Without this system, the onslaught of DSBs from replication fork collapse, oxidative damage, ambient radiation, and other sources would severely limit any cell lineage. Moreover, the weakening of genomic integrity would make multicellular organisms more susceptible to cancer [1] and probably accelerate the aging process [2,4,34,36,37]. The multiplicity of pathways available for DSB repair provides more than mere redundancy: each pathway comes with its own set of advantages and risks. NHEJ, for example, has the advantage of fewer prerequisites than HR-h or SSA, since it does not require a matching template or a flanking duplication. Its disadvantage, however, is that it usually results in base additions, deletions, or substitutions at the site of repair. At the other extreme is HR-s, which provides minimal risk of mutational changes following repair. A major disadvantage of HR-s, however, is that it is only available following replication. Also, its requirements for homology search and extensive DNA synthesis might be too time-consuming under some circumstances. The wide array of DSB repair methods available to the cell provides the flexibility to handle a variety of situations in an optimal way. We obtain a fuller picture of the entire apparatus by analyzing DSB repair mutations in an experimental system that provides accurate quantitative measurements of multiple outcomes. In particular, we can learn not only how each mutation reduces certain outcomes, but also how the system as a whole compensates for the defect by making increased use of alternative pathways.

## Materials and Methods

### Measurements of DSB repair outcomes with Rr3.

Crossing and scoring procedures were as described previously [28,34–36] except for the presence of repair mutations. Rr3 was on Chromosome 2, position 48C, for all experiments, but the ubiquitin-driven I-SceI endonuclease transgene was at one of three locations depending on which repair mutation(s) were also present. Earlier results have shown that different endonuclease locations can result in subtle differences in the DSB repair outcome frequencies [28]. Therefore, all comparisons were done between mutant genotypes and controls with the same endonuclease source.

All DSB repair measurements pertain to the premeiotic germline of the test males whose progeny were scored. These males were always mated within two weeks of when they eclosed to avoid differences related to male age [34].

### Generation of deletion mutations at *lig4* and *ku70* loci.

We mobilized nearby P element insertions to generate flanking deletions in the areas of *lig4* and *ku70.* Details of the procedure and the resulting deletion alleles are in Figure S1.

### Statistical methods.

Each estimate of a DSB repair outcome frequency reported in Table 1 is the average of the indicated number of independent estimates from individual test males. The standard errors given in Table 1 and shown in Figures 3–5 were computed from these replicates. These standard errors are provided as a rough calibration of the precision of each estimate, but they were not used for the hypothesis tests in which mutant genotypes are compared with controls. Instead, we used the multiple independent replicates for each estimate in permutation tests [50], which do not rely on assumptions of normality. Further details of how these permutation tests were performed are published elsewhere [35].

## Supporting Information

Figure S1 Deletion Mutations at *ku70* and *lig4* Loci (118 KB PDF)

Figure S2 Recombination Frequencies in the Absence of SSA (91 KB PDF)

Figure S3 Mutagen Sensitivity of *ku70* Mutants and Rescue by Transgene (137 KB PDF)

Figure S4 Sensitivity of *ku70* Mutants to Gamma Radiation and Transgene Rescue (141 KB PDF)

Figure S5 Bristle Phenotype of *ku70^EX8^* Mutants and Rescue by Transgenes (87 KB PDF)

## Abbreviations

DSB: double-strand break
HR: homologous recombination repair
HR-h: homologous repair from the homolog
HR-s: homologous repair from the sister chromatid
NHEJ: nonhomologous end-joining
NHEJΔ: NHEJ with long deletion
Rr3: repair reporter construct
SSA: single-strand annealing
UIE: ubiquitin-driven I-SceI endonuclease
