# Supplementary material for: Multiple-Pathway Analysis of Double-Strand Break Repair Mutations in Drosophila
Source: PLoS Genet. 2007 Apr 13;3(4):e50. doi: 10.1371/journal.pgen.0030050 (PMC1851981; doi:10.1371/journal.pgen.0030050)
Supplement: Supplementary file 1 [file pgen.0030050.sg001.pdf]

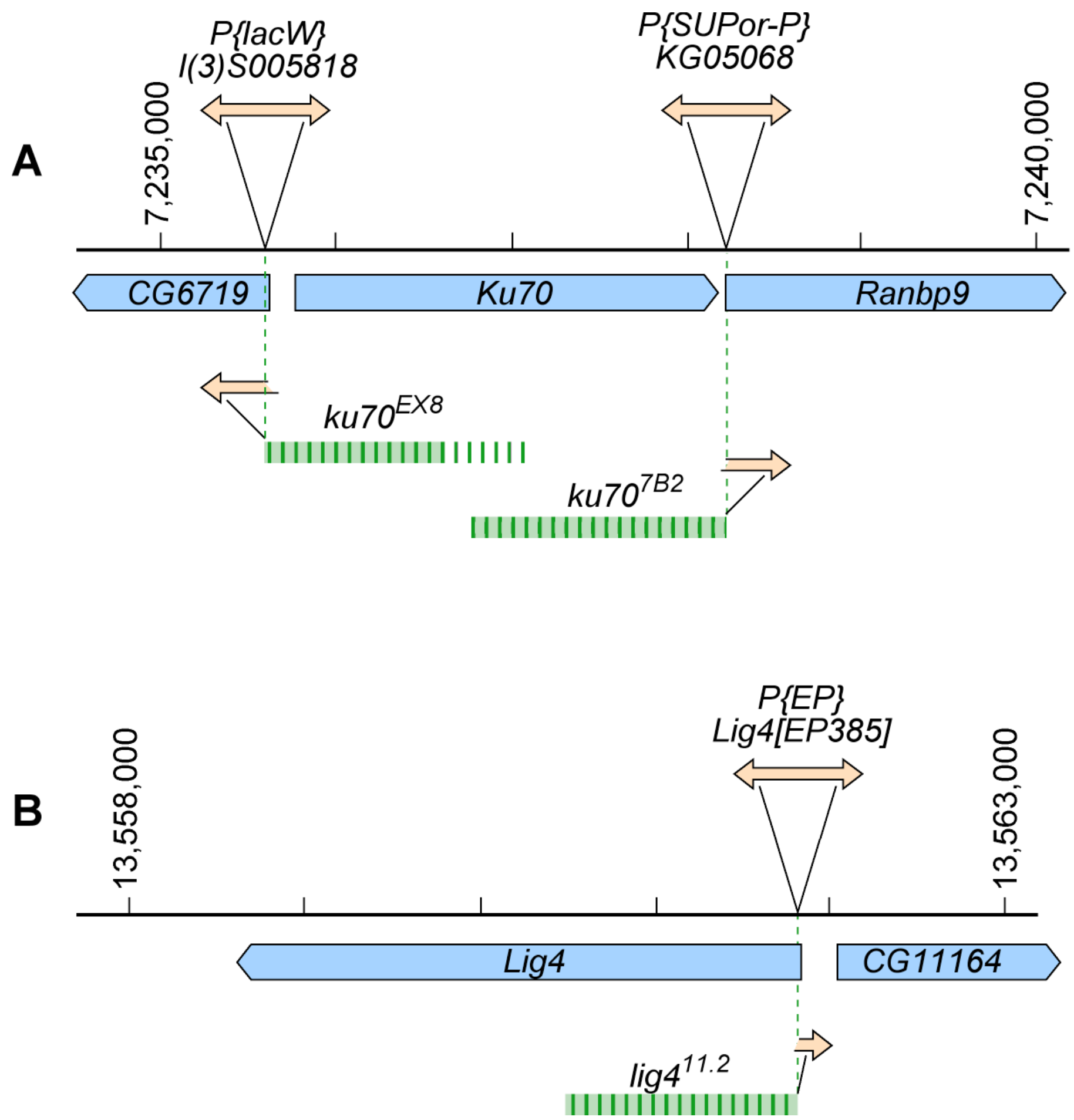

Figure S1. Deletion mutations at *ku70* and *lig4* loci. Deletions at both loci were obtained by mobilization of a nearby P element insertion and selection for loss of the *white*$^+$ marker carried within the element. Maps and coordinates are from FlyBase [82] release 5.1. **(A)** Map of the vicinity of *Ku70* showing the two insertions used to generate deletions. The position of the *P{lacW}* element was refined with TAIL PCR (Liu, YG and RF Whittier, 1995. Genomics 25: 674) to obtain flanking sequence information indicating its location at 7235543. The deletions, shown as green barred lines, each had one breakpoint within the P element and the other within the *Ku70* gene. The endpoints of *ku70*^{EX8} were estimated from amplicon sizes, with the unshaded portion representing the area of uncertainty. Approximately 1kb of the *P{lacW}* element remains. The limits of *ku70*^{7B2} were obtained by sequencing across the breakpoint to obtain: *GCAGCTATCG AACTGGACGC CTTCCAGG TG*gttaatcc cctt**CCCATT GGTTAATCAG CAGACCCTCG TTGGCGTAAC GGAA**. Bases within *Ku70* are italicized, those derived from the *white*$^+$ region of the P element are in boldface, and bases of unknown origin are shown as lower case. **(B)** Map of the vicinity of *lig4* showing the P insertion used to generate deletion *lig4*$^{11.2}$. The sequence at the deletion breakpoint is: *TTTCTGCAAT TTGTACGCC CGCTGAATG****T*** **ATGTTATTT CATCATG***AT CACTGTGAA CATCTGTTC AAGCAATGT GACC*, where bases within *Lig4* or to the right of the insertion are italicized and the P element bases are in boldface.
