# Supplementary material for: Multiple-Pathway Analysis of Double-Strand Break Repair Mutations in Drosophila
Source: PLoS Genet. 2007 Apr 13;3(4):e50. doi: 10.1371/journal.pgen.0030050 (PMC1851981; doi:10.1371/journal.pgen.0030050)
Supplement: Supplementary file 2 [file pgen.0030050.sg002.pdf]

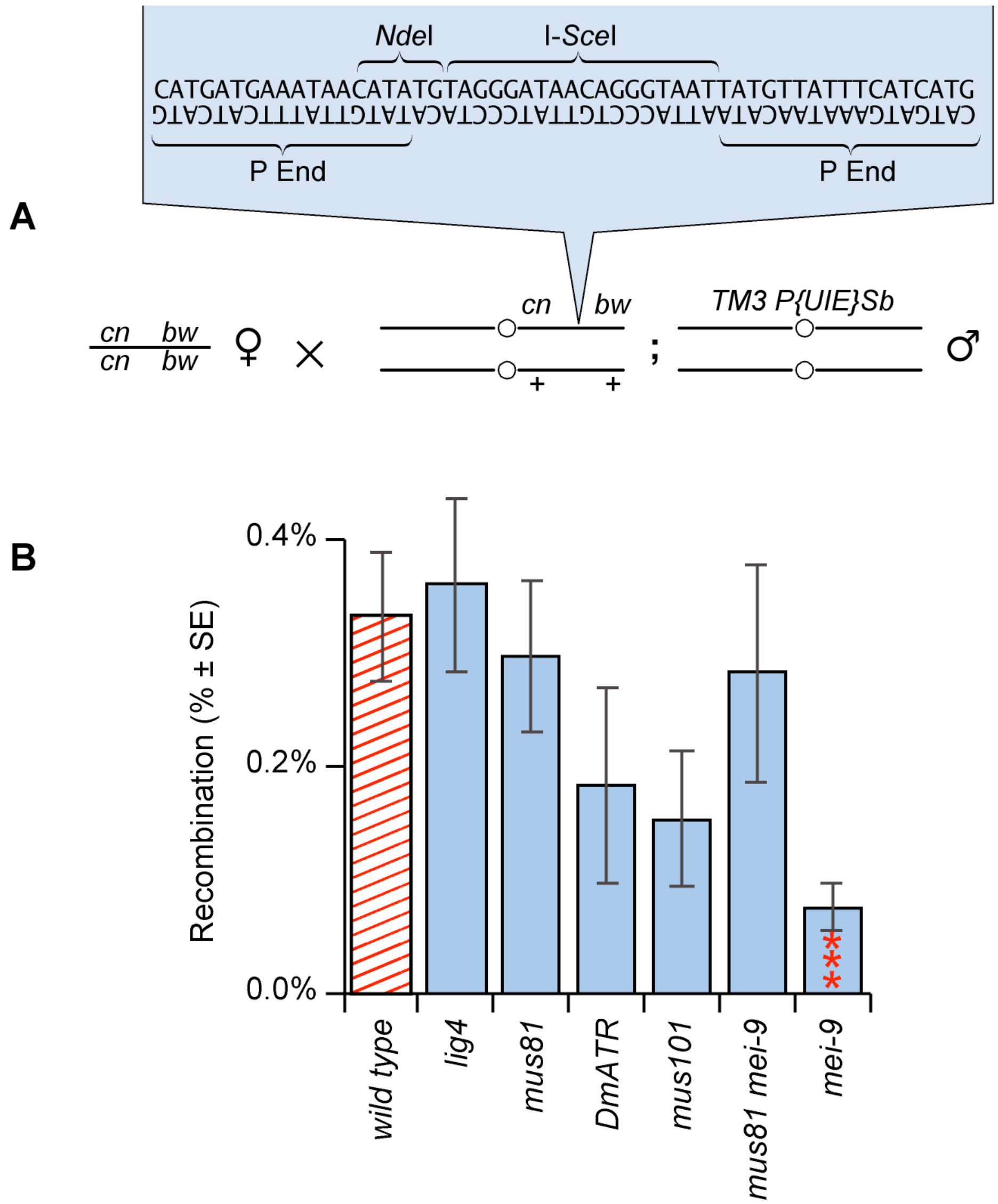

Figure S2. Recombination frequencies in the absence of SSA. (A) Crossing over was measured in the premeiotic germ cells of the males shown by scoring offspring for the visible markers *cinnabar (cn)* and *brown (bw)*. DSBs are made at the I-*Sce*I cut site on chromosome 2 [35]by endonuclease expressed on chromosome 3 [28]. **(B)** Recombination frequencies and standard errors were obtained from multiple independent single-male replicates. The numbers of replicates, from left to right, were 134, 67, 83, 27, 67, 54 and 129. The total numbers of offspring scored were 18354, 8836, 12187, 2405, 7748, 4289 and 19300. Statistical significance was assessed with two-tailed permutation tests similar to those used with Rr3 measurements (see Materials and Methods). Each mutant genotype was compared with the wildtype controls.
