# Supplementary material for: Multiple-Pathway Analysis of Double-Strand Break Repair Mutations in Drosophila
Source: PLoS Genet. 2007 Apr 13;3(4):e50. doi: 10.1371/journal.pgen.0030050 (PMC1851981; doi:10.1371/journal.pgen.0030050)
Supplement: Supplementary file 3 [file pgen.0030050.sg003.pdf]

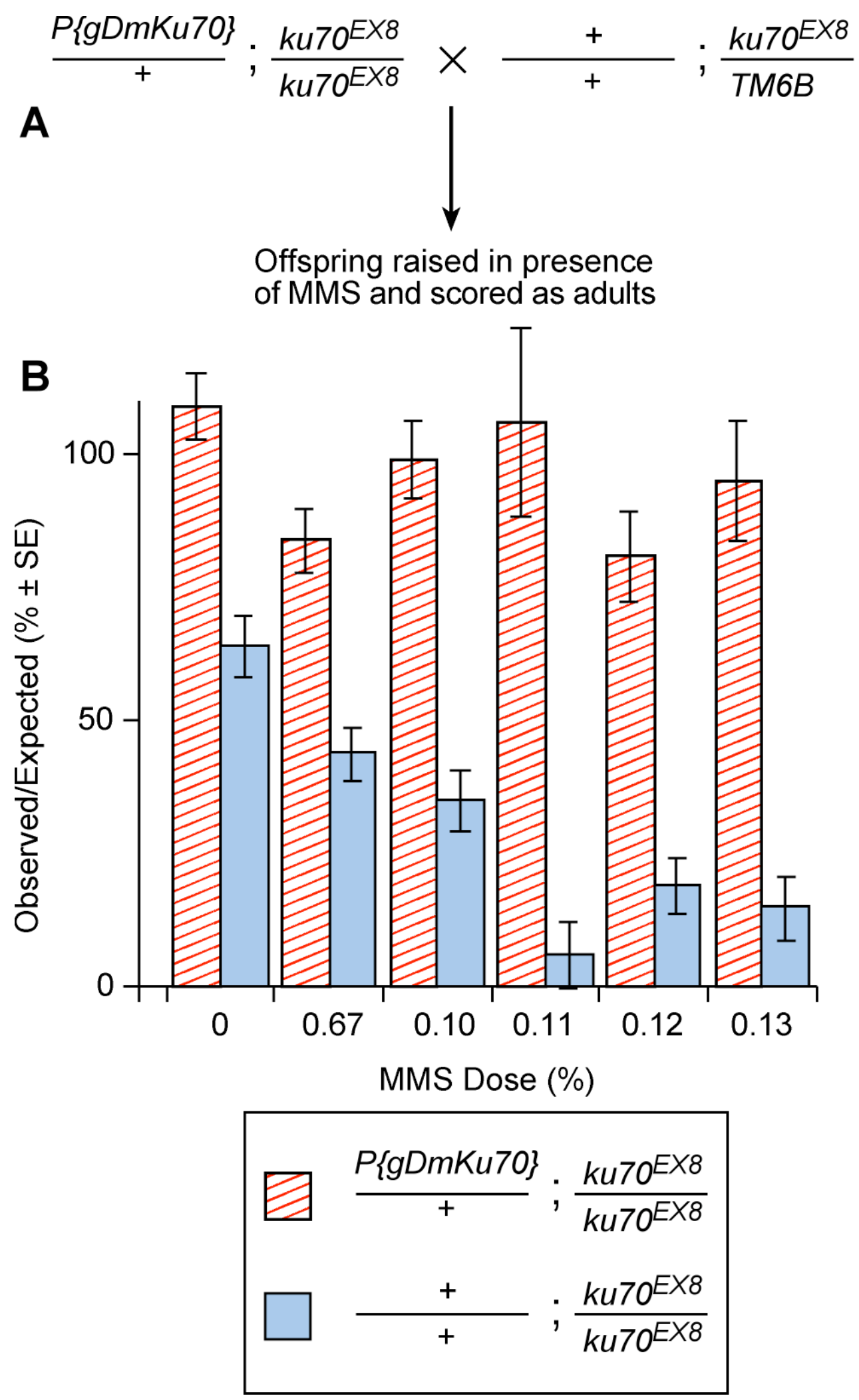

Figure S3. Mutagen sensitivity of *ku70* mutants and rescue by transgene. Results show that *ku70*^{EX8} homozygotes had decreased survival in the presence of a chemical DSB-forming agent relative to their heterozygous controls. The *Ku70* transgene restored normal sensitivity. Survival of *ku70*^{EX8} homozygotes lacking the transgene was reduced even in the absence of the mutagen. **(A)** Offspring from the cross shown were treated with methyl methanesulfonate (MMS) as described [65,68] and scored for the presence of the transgene, *P{gDmKu70}13* on chromosome 2 and the balancer (*i.e.,* dominantly marked crossover suppressor), *TM6B*, on chromosome 3. This transgene carries both *Ku70* and CG6719 (Fig. S1). **(B)** Ratios of the observed to expected numbers of surviving offspring. Expected numbers were half the numbers of surviving *TM6B* heterozygotes. No formal hypothesis tests were performed, but approximate standard errors were computed from the binomial distribution. We assumed that there was a fixed number of *ku70*^{EX8} homozygous embryos with and without the transgene equal to the number of surviving *TM6B* heterozygotes. That is, we used $SE = 2\sqrt{k(t-k)/t^3}$ where k is the number of *ku70*^{EX8} surviving homozygotes and t is the number of *TM6B* heterozygous survivors.
