# Supplementary material for: Multiple-Pathway Analysis of Double-Strand Break Repair Mutations in Drosophila
Source: PLoS Genet. 2007 Apr 13;3(4):e50. doi: 10.1371/journal.pgen.0030050 (PMC1851981; doi:10.1371/journal.pgen.0030050)
Supplement: Supplementary file 4 [file pgen.0030050.sg004.pdf]

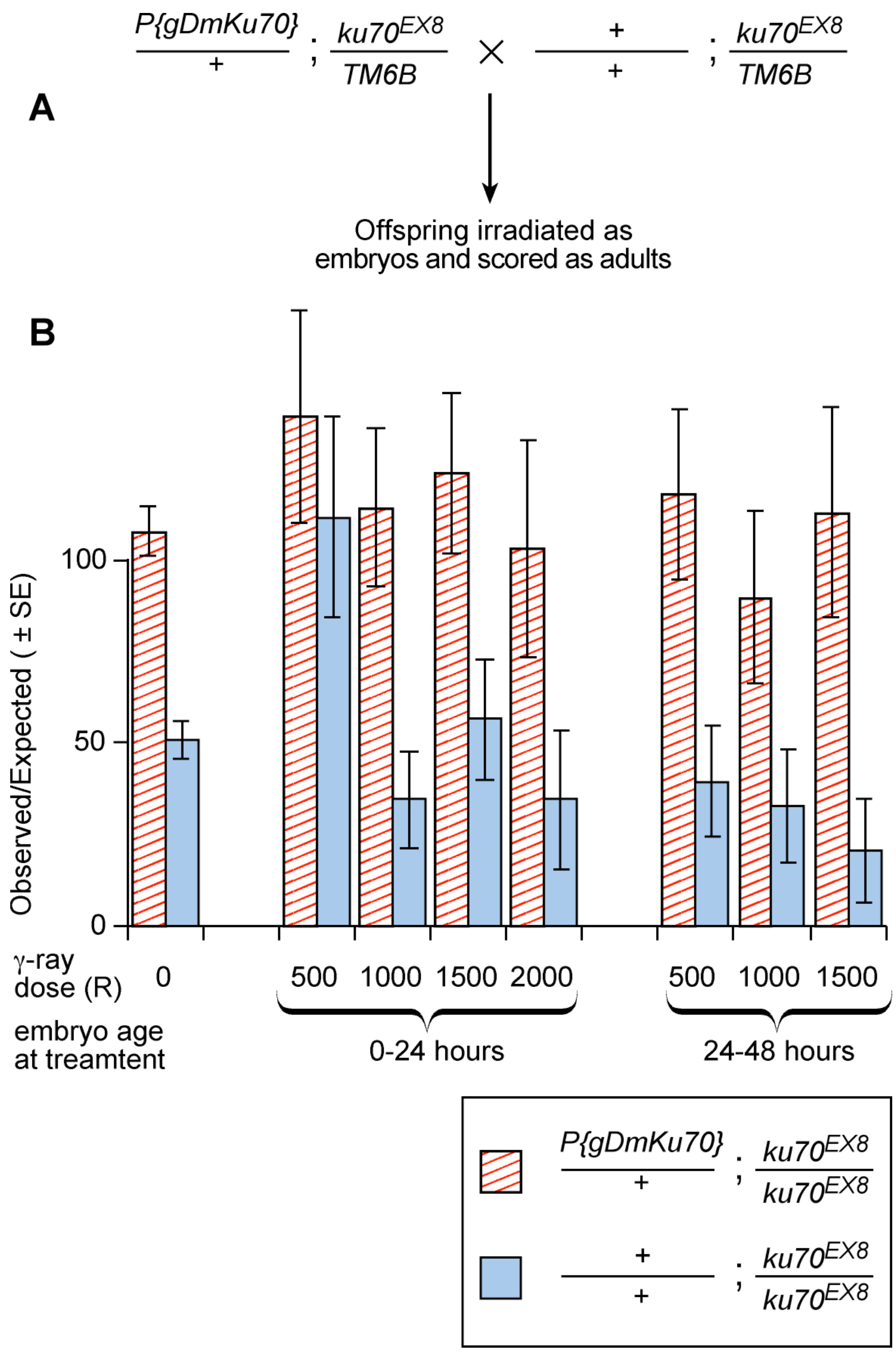

Figure S4. Sensitivity of *ku70* mutants to gamma radiation and transgene rescue. Results suggest that homozygous *ku70* mutants are mildly hypersensitive to γ radiation but are fully rescued by the *P{gDmKu70}13* transgene. **(A)** Embryos from the cross shown were exposed to a ^{137}Cs source during the first or second day after eggs were laid. Survivors were then scored as in Fig. S3. **(B)** Observed/expected ratios were computed as in Fig. S3 except that the expected numbers were one-fourth of the total *TM6B* heterozygotes. The factor of 1/4 instead of 1/2 arises because *TM6B* is heterozygous in both parents. No formal hypothesis tests were computed in the absence of independent replicates, but approximate standard errors were calculated se $SE = 4\sqrt{k(t-k)/t^3}$ under the same assumptions as in Fig. S3.
