# Supplementary material for: Multiple-Pathway Analysis of Double-Strand Break Repair Mutations in Drosophila
Source: PLoS Genet. 2007 Apr 13;3(4):e50. doi: 10.1371/journal.pgen.0030050 (PMC1851981; doi:10.1371/journal.pgen.0030050)
Supplement: Supplementary file 5 [file pgen.0030050.sg005.pdf]

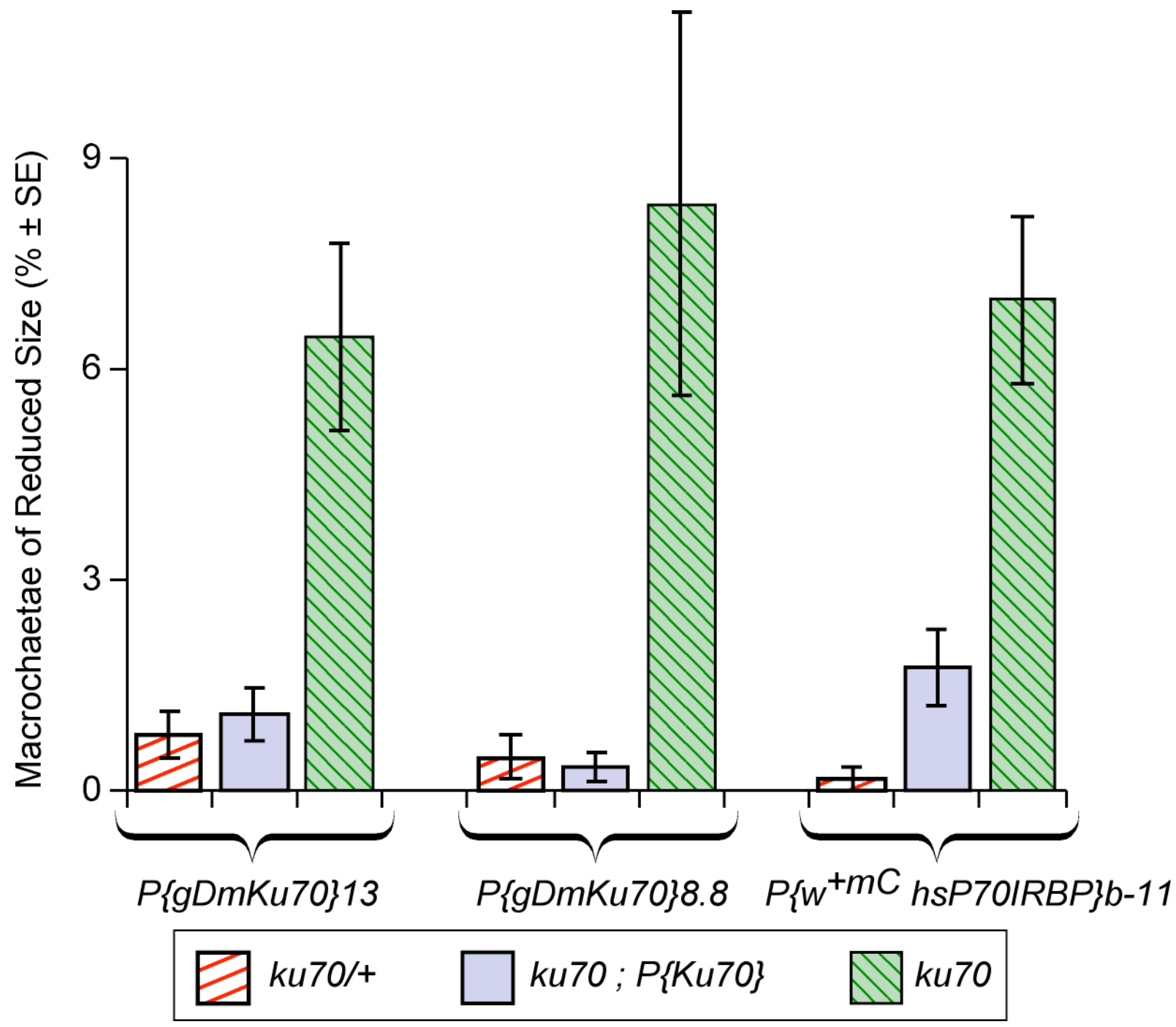

Figure S5. Bristle phenotype of *ku70^{EX8}* mutants and rescue by transgenes. Flies mutant for *ku70* have an excess number of thoracic bristles which are deformed or reduced in size. This effect was rescued by *P{Ku70}* transgenes. Three sets of crosses were performed, each with a different transgene construct. From each cross, offspring were classified as heterozygous controls (*ku70^{EX8}*/+*)*, homozygous mutants bearing the transgene, or homozygous mutants without the transgene. Offspring were then separated and coded so that the scorer would not know their genotype. They were then examined for size changes in the twelve major thoracic macrochaetae. Standard errors were computed by treating each fly as an independent estimate. The numbers of bristles scored (left to right) were 648, 660, 372, 444, 672, 144, 672, 684 and 660. Two of the transgenes (*P{gDmKu70)13* and *P{gDmKu70}8.8*) are in reference [38] and the third is in [22]. The first two also carry the CG6719 gene (see Fig. S1) but the third does not. Since all three rescued the bristle phenotype, we conclude that *ku70* is responsible for the increased number of deformed and reduced bristles.
